# Kinesio Taping Relieves Pain and Improves Isokinetic Not Isometric Muscle Strength in Patients with Knee Osteoarthritis—A Systematic Review and Meta-Analysis

**DOI:** 10.3390/ijerph181910440

**Published:** 2021-10-04

**Authors:** Hsin-Yu Mao, Meng-Tzu Hu, Yea-Yin Yen, Shou-Jen Lan, Shin-Da Lee

**Affiliations:** 1 Department of Health Care Administration, Asia University, Taichung 413305 Taiwan; hsinyumao@hotmail.com; 2 Department of Physical Therapy, Shu-Zen Junior College of Medicine and Management, Kaohsiung 82144, Taiwan; 3 Department of Physical Therapy, Tzu-Hui Institute of Technology, Pingtung 926001, Taiwan; hmt0704@yahoo.com.tw; 4 School of Medical Science, Putian University, Putian 351100, China; yyyen0302@gmail.com; 5 Department of Physical Therapy, Asia University, Taichung 41354, Taiwan; 6 Department of Physical Therapy, China Medical University, Taichung 406040, Taiwan; 7 School of Rehabilitation Medicine, Weifang Medical University, Weifang 261000, China

**Keywords:** kinesiology tape, degenerative joint disease, pain, isokinetic torque

## Abstract

This study investigated the effects of kinesio taping (KT) or KT plus conventional therapy on pain, muscle strength, funrefction, and range of motion in patients with knee osteoarthritis (OA). Data sources: Databases included PubMed, Ovid Medline, CINAHL, Airiti Library, EMBASE, and WOS search engines. Search terms related to KT and knee OA were combined and searched. Articles that met the inclusion criteria and were graded with a Jadad score ≥3 were included in a meta-analysis to calculate the total effect. The exclusion criteria were non-English-language articles, non-original articles, non-full-text articles, no description of the intervention, or articles with a Jadad score ≤2. Eleven articles were included in the meta-analysis. KT treatment had a significant small total effect on pain reduction (*p* < 0.001; *n* = 1509; standardized mean difference (SMD) = −0.42; 95% CI = −0.65 to −0.18) and a significant moderate total effect on isokinetic muscle strength improvement (*p* = 0.001; *n* = 447; SMD = 0.72; 95% CI = 0.28 to 1.16). No significant total effects of KT on isometric muscle strength, time to complete functional tasks, or ROM improvement were found. KT or KT plus conventional therapy has a significant effect on pain relief and isokinetic but not isometric muscle strength improvement in patients with knee OA. KT can be an effective tool for treating knee OA pain and is especially valuable for aiding in isokinetic muscle strength. (PROSPERO register ID: CRD42021252313)

## 1. Introduction

Knee osteoarthritis (OA) is a chronic progressive disease commonly seen in the elderly. The main symptoms include pain, joint stiffness, reduction in the range of motion (ROM), crepitus during activity, inflammation and swelling. Furthermore, patients with knee OA may suffer from joint deformity, muscle atrophy of the lower extremities, abnormal gait, or even ambulatory inability [1]. As the quality of life of knee OA patients remains very poor, the pain reduction, muscle strength enhancement, deformity prevention, and function improvement of knee OA are important issues in the aging population [1,2].

Common treatments of knee OA include lifestyle adjustments, physical therapy, anti-inflammatory medications, intra-articular injections, and knee arthroplasty [1]. The usage of kinesio tape (KT) was regarded as an intervention or as a supplementary treatment for patients with knee pain, which has become popular [3,4]. KT, developed by Dr. Kenzo Kase, was characterized by its specific thickness and high elasticity, as well as its capability of stretching up to 130–140% of its resting static length, ensuring free mobility of the applied muscle or joint [5]. Most studies agreed that KT can reduce pain by stimulating cutaneous mechanoreceptors and increasing afferent feedback [3,6,7]. Another goal of KT is to enhance muscle performance and further improve athletic performance [5,8]. Such characteristics can also help patients with muscle weakness and functional disability [6]. Patients with knee OA often suffer from muscle weakness in the lower extremities. Pain, joint inflammation, and joint swelling can lead to arthrogenic muscle inhibition (AMI), leading to muscle atrophy and a decrease in muscle strength [6]. Dr. Kenzo Kase claims that KT applied from the origin to insertion of the muscle can aid in muscle contraction [5]. However, the controversial effects of KT on muscle strength have shown conflicting results [8,9]. Murray reported that KT application enhanced electromyographic activities in the quadricep muscles during the postoperative phase of anterior cruciate ligament repair [10]. In contrast, Lins et al. reported that KT application to the quadriceps was not capable of altering lower limb function, one-footed static balance, or peak knee extensor torque in healthy women [11].

Several systematic reviews and meta-analyses of KT or non-elastic taping treatment in different knee or musculoskeletal problems have been published in recent years. A meta-analysis by Parreira et al. found that the therapeutic effects of KT were no better than those of the sham-taping groups or comparison groups treated with conventional therapy in pain intensity, disability, quality of life, return to work, and global impression of recovery in different musculoskeletal problems [12]. A systematic review by Logan et al. indicated that support taping (KT or McConnell tape) could reduce pain as an adjunct to traditional exercise therapy in patients with patellofemoral pain syndrome [13]. Another meta-analysis by Chang et al. compared the effect of KT versus McConnell tape in patients with patellofemoral pain syndrome and reported that using KT has a significant effect on pain reduction, motor function improvement and muscle activity change [14]. Two meta-analyses analyzed the effect of KT on muscle strength. Yam et al. found that KT was superior to controls for improving lower limb muscle strength in individuals with muscle fatigue or chronic musculoskeletal problems, but not in populations without disability [15]. On the other hand, Lu et al. did not find a significant difference between the KT and control groups regarding quadricep muscle strength in knee OA patients [16]. Controversial results might be derived from inconsistencies in study design, methodologies of KT application, study populations, or instruments used to measure outcomes.

To date, no systematic review has provided a complete summary and meta-analysis of the existing literature on the effects of KT in treating knee OA patients, especially focusing on muscle strength improvement, and analyzing muscle strength with isokinetic and isometric measurements separately. Therefore, the aim of this study was to investigate the effect of KT or KT plus conventional therapy on pain, muscle strength, function, and ROM in patients with knee OA or those who had undergone total knee replacement (TKR) due to severe OA. Moreover, collecting and analyzing the data of interventions using KT as the primary treatment or in addition to rehabilitation will provide a deeper understanding of the therapeutic mechanism of KT and help practitioners to make clearer clinical decisions.

## 2. Materials and Methods

### 2.1. Data Sources and Search Strategy

The existing literature was systematically searched from Jan 1970 to March 2020 using the PubMed, Ovid Medline, CINAHL, Airiti Library, EMBASE, and WOS databases. Search terms related to KT (kinesio tape, kinesio taping, kinesiology tape, kinesiology taping, KT tape, and kinesiotaping) were used in combination with terms to identify interventions for knee OA or TKR (knee osteoarthritis, knee OA, and total knee replacement). This systematic review was registered in PROSPERO (CRD42021252313) where the review protocol can be accessed.

Since the present study is a systematic review and meta-analysis of the previously published literature, ethical approval or signing of written consent was not required.

### 2.2. Inclusion Criteria

Studies that met the following criteria were included: 1. the study employed a design for comparative analysis; 2. the participants were adults with a diagnosis of knee OA or post-TKR due to severe osteoarthritic changes in the knee; 3. the interventions of interest were kinesio taping alone or kinesio taping in addition to other conventional therapies, including physical therapy, rehabilitation or medicine; 4. the outcomes of interest were pain, muscle strength, functional performance, and ROM; and 5. there were sufficient data to calculate the standard mean difference (SMD).

### 2.3. Exclusion Criteria

Articles with any of the following criteria were excluded: 1. non-English-language articles, review articles, meta-analyses, editorials, letters, comments, conference abstracts or case reports; 2. duplicate or non-full-text articles; 3. articles that did not describe an intervention trial; and 5. articles with poor quality according to a Jadad score ≤ 2.

### 2.4. Screening

Two reviewers, each with more than ten years of experience in physical therapy, screened all articles to identify those that met the study criteria. The methods, evaluations, and results of the articles were collected, and the outcomes of kinesio taping were analyzed. Finally, the Jadad quality score was used to grade the quality of the articles. The Jadad scale contains five items that are graded on a 5-point scale to assess the methodological quality of an article (randomization, blinding, and an account of all patients), and an article with a Jadad score ≥ 3 was considered to be of good quality [17].

### 2.5. Data Collection and Meta-Analysis

The extracted data from the included articles were recorded, and a meta-analysis was performed with MedCalc software v18.2.1 (MedCalc, Ostend, Belgium). If the outcomes were evaluated at multiple time points during the intervention, only the data at the endpoint of the treatment were used to compare with the baseline data. The means and standard deviations of continuous variables in the articles were analyzed to estimate the SMD and 95% confidence interval (CI). Heterogeneity was measured with the Cochran Q test, and the results were considered statistically significant when *p* < 0.05 or I^2^ > 50%. A file drawer analysis was used to explore publication bias. A total effect was calculated by a total random-effects model to determine the outcome effects of KT when significant heterogeneity was present; in contrast, a fixed-effects model was used when no significant heterogeneity was present. The SMD and 95% CI of the outcome variables of individual studies and the total effects are presented in forest plots. The effect size was graded as very small (SMD = 0.01~0.2), small (SMD = 0.2~0.5), moderate (SMD = 0.5~0.8), or large (SMD > 0.8), as suggested by Cohen [18].

## 3. Results

### 3.1. Search Results

**Figure 1:**
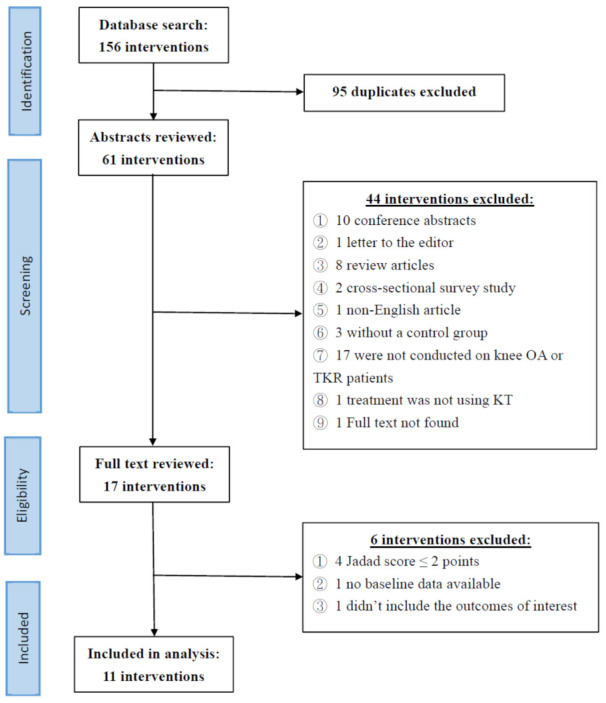
Flowchart of literature search (OA = osteoarthritis; TKR = total knee replacement; KT = kinesio tape).

The initial database search resulted in the identification of 156 interventions. After the exclusion of 95 duplicates, 61 interventions were left, and the abstracts of these articles were reviewed. In accordance with the exclusion criteria, 44 of the 61 articles were excluded for reasons including study type, study design, non-English articles, diagnosis of participants, or unavailability of full text and left 17 articles. After reviewing the full text of these 17 articles, six more studies were excluded due to low quality (Jadad score ≤ 2), insufficient data, or not including the outcomes of interest. Finally, 11 articles were included in the present meta-analysis. The precise process of the literature search and screening is shown in the flowchart in Figure 1. The details of the 11 studies included in the meta-analysis are listed in Table 1.

### 3.2. Characteristics of Included Studies

In total, the included 11 RCTs reflected data from 739 subjects, comprising 564 women (76.32%) and 175 men (23.68%) (Table 1). Among the eleven studies, only the participants (9%) in the study by Donec and Krisciunas were diagnosed post-TKR patients due to severe knee OA [4], while the participants in the other ten studies (91%) were all patients diagnosed with knee OA (Table 1). Regarding the intervention, four of the eleven studies (36%) used KT in conjunction with conventional therapy in the experimental group compared with a control group receiving either only conventional therapy or conventional therapy with sham tape. The other seven studies (64%) compared a pure KT treatment with placebo kinesio taping or other tapes without elasticity. The method of taping in the experimental group in all studies followed Dr. Kase’s principles, but they were not all applied in an identical manner. Nine studies used a Y- or I-strip KT applied from the origin to the insertion of the rectus femoris (RF) muscle for facilitation and applied tension varying from 15% (paper-off tension) to 70%. Rahlf et al. used an I-strip KT with maximum tension over the patella, from the tibial tuberosity to one-third of the RF muscle [27]. Wageck et al. used a muscle relaxation technique on the RF to minimize pressure between the femur and patella rather than a facilitation technique [22]. Some studies also added other KT strips to achieve a better therapeutic effect. Anandkumar et al. and ÖĞÜT et al. added I- or Y-strips on the vastus medialis and vastus lateralis muscles to facilitate the whole quadricep muscle [6,25]. In two studies, KT techniques were applied on the hamstrings for facilitation, while two other studies added fan strips to promote lymphatic reflux. Three studies added I-strips to stabilize the medial and lateral collateral ligaments of the knee, and another study stated that an I-strip was applied mediolaterally over the patella without explaining its purpose. Wageck et al. used star-shaped KT to relieve pain [9]. Moreover, the post-treatment evaluation time ranged from immediately (five of eleven studies, 45%) to 6 weeks after KT application, and four studies (36%) conducted a follow-up evaluation.

### 3.3. Outcomes

#### 3.3.1. Pain

**Figure 2:**
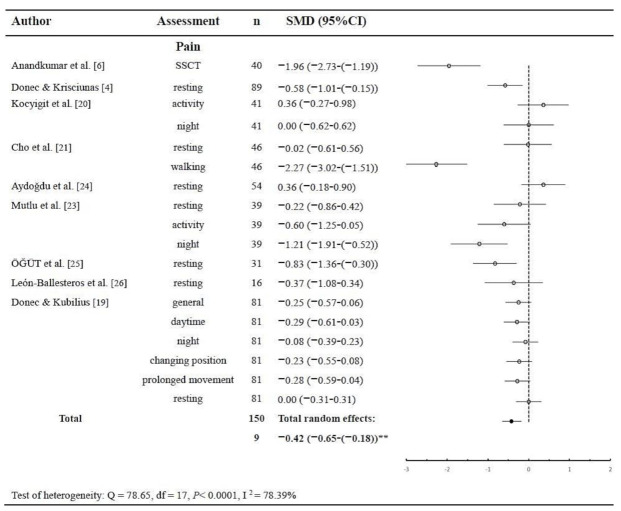
Pooled estimate of effects on pain with kinesio taping ** *p* < 0.05 (*n* = number of subjects; SMD = standard mean difference; CI = confidence interval; SSCT = standardized stair climbing task; df = degree of freedom).

Knee pain during different activities or under various circumstances, such as pain during the standardized stair climbing task (SSCT), resting pain, activity pain night pain, and general pain, were measured by the visual analog scale (VAS) or numeric pain rating scale (NPRS) in nine studies with a total number of subjects of 1509 (82%). Significant heterogeneity was found for pain change (*p* < 0.0001; Q = 78.65; DF = 17; I^2^ = 78.39%; 95% CI = 66.38 to 86.10). There was a significant small total effect of pain relief for knee OA patients treated with KT (*p* < 0.001; *n* = 1509; SMD = −0.42; 95% CI = −0.65 to −0.18) (Figure 2). A negative SMD indicates better pain relief for the experimental group.

#### 3.3.2. Muscle Strength

**Figure 3:**
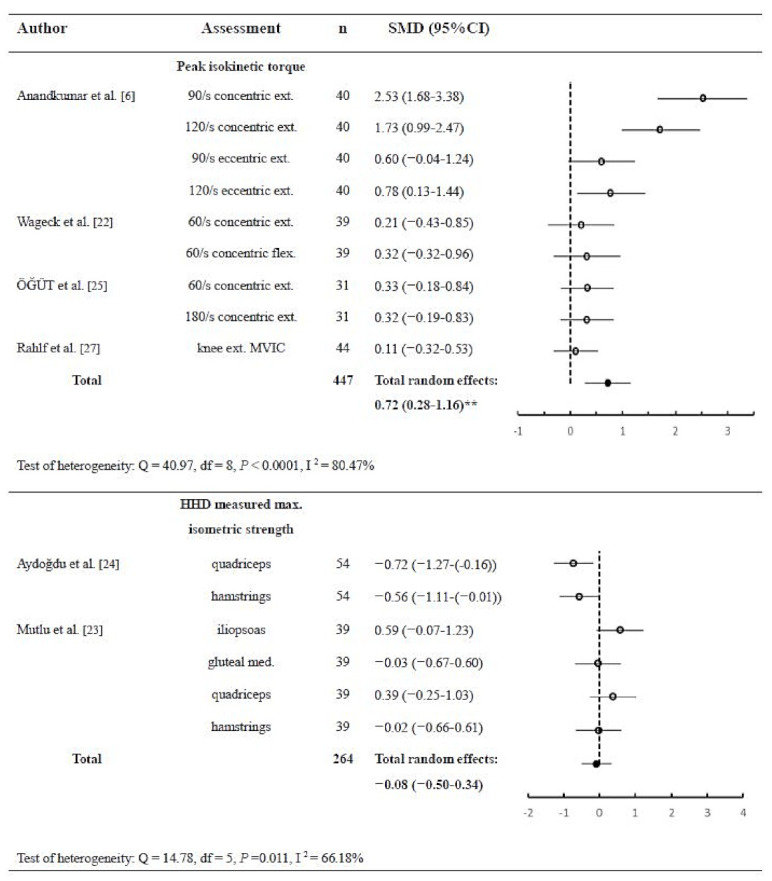
Pooled estimate of the effects of kinesio taping in peak isokinetic torque and maximal isometric strength measured by HHD ** *p* < 0.05 (*n* = number of subjects; SMD = standard mean difference; flex. = flexion; ext. = extension; MVIC = maximal voluntary isometric contraction; df = degree of freedom; HHD = handheld dynamometer).

Muscle strength was measured by using an isokinetic machine or a handheld dynamometer (HHD) in six studies (55%). An isokinetic machine can assess the concentric or eccentric peak isokinetic torque generated by knee flexors or extensors under different angular speeds or maximal voluntary contraction, whereas an HHD measured the isometric force. According to different assessment methodologies, in the muscle strength analysis, the studies were divided into two subgroups: the isokinetic machine and HHD subgroups. Four studies with nine measurements were included in the meta-analysis of the isokinetic machine subgroup. Although these measurements showed significant heterogeneity (*p* < 0.0001; Q = 40.97; DF = 8; I^2^ = 80.47%; 95% CI = 63.77 to 89.48), a significant moderate total effect favoring KT treatment was found (*p* = 0.001; *n* = 447; SMD = 0.72; 95% CI = 0.28 to 1.66). On the other hand, in the HHD subgroup, two studies with six measurements were included and showed heterogeneity (*p* = 0.0113; Q = 14.78; DF = 5; I^2^ = 66.18%; 95% CI = 19.13 to 85.85) with a non-significant total effect (*p* = 0.705; *n* = 264; SMD = −0.08; 95% CI = −0.50 to 0.34) (Figure 3).

#### 3.3.3. Function

**Figure 4:**
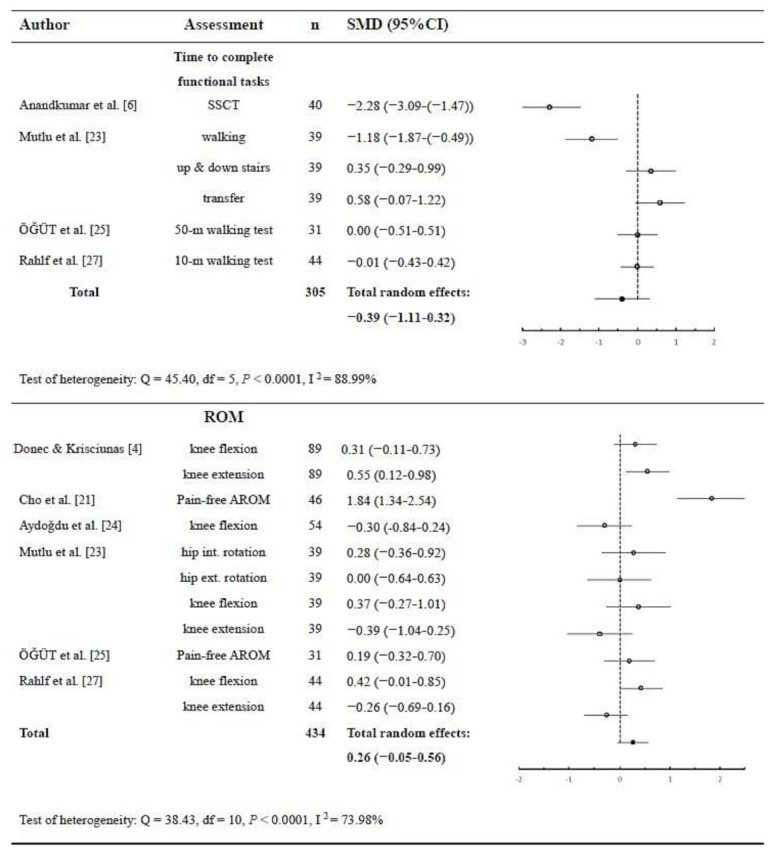
Pooled estimate of the effects of kinesio taping in completing functional tasks and range of motion (*n* = number of subjects; SMD = standard mean difference; SSCT = standardized stair climbing task; ROM = range of motion; AROM = active range of motion; int. = internal, ext. = external; df = degree of freedom).

Function was measured according to the time used to accomplish functional tasks, such as ascending and descending stairs, walking for a certain distance, and travelling a distance to reach a chair and sit down. A shorter time to accomplish functional tasks represented better functional performance. Results showed significant heterogeneity (*p* < 0.0001; Q = 45.40; DF = 5; I^2^ = 88.99%; 95% CI = 78.65 to 94.32), and a non-significant total random effect was found (*p* = 0.282; *n* = 305; SMD = −0.39; 95% CI = −1.11 to 0.32) in the four studies that measured six functional tasks, indicating that the participants did not perform the tasks faster than those without KT application (Figure 4).

#### 3.3.4. ROM

ROM before and after treatment was measured with a goniometer in six studies (55%), providing data of eleven ROMs in the lower extremity. Meta-analysis showed significant heterogeneity (*p* < 0.0001; Q = 38.43; DF = 10; I ^2^ = 73.98%; 95% CI = 52.65 to 85.70) and a non-significant total random effect (*p* = 0.10; *n* = 669; SMD = 0.26; 95% CI = −0.05 to 0.56) (Figure 4).

## 4. Discussion

The present study is the first systematic review and meta-analysis to investigate the effect of KT on isokinetic and isometric muscle strength separately in knee OA populations. The main finding of this study is that KT had a significant small total effect on pain relief and a significant moderate total effect on isokinetic muscle strength improvement in knee OA patients. It was important to include studies that used KT as an adjunct therapy as well as those that used KT as a main treatment. Knee taping combined with exercise provides superior outcomes compared with exercise alone, and a complete rehabilitation program is multifactorial [13]. Seventy-six percent of the sample in the study were female, which is close to the gender proportion of knee OA prevalence in the population [28].

### 4.1. Significant Effect in Pain Relief

For knee OA patients, pain is the most invasive symptom, which not only affects daily activities but also causes muscle atrophy due to AMI [6,29]. The effect of KT on reducing pain has been proven in various knee problems. Balki, Göktaş, and Öztemur’s study found that KT significantly reduced resting pain, night pain, and activity pain of patients in the acute phase of anterior cruciate ligament reconstruction compared with the sham-tape control group [29]. Rahlf, Braumann, and Zech used KT on knee OA patients, and a significant effect in the Western Ontario and McMaster Universities Osteoarthritis Index (WOMAC) pain subscale was found in the KT-treated intervention group compared with the sham-tape and no-tape control groups [27]. Findings of the present study on pain reduction are consistent with the previous literature [14,27,29]. The most widely accepted explanation for this pain reduction effect is gate control theory, in which the tape stimulates neuromuscular pathways via increased afferent feedback, and this increase in afferent stimuli of large-diameter nerve fibers can serve to block the input received from the small-diameter nerve fibers conducting nociception, resulting in pain relief [11].

### 4.2. Significant Effect on Isokinetic Muscle Strength Improvement

Muscle weakness of the lower extremity is common in patients with knee pain and is often observed bilaterally [30]. Quadricep training was considered as one of the most important components of a rehabilitation program as the quadricep muscle can stabilize the knee and further reduce knee pain [1]. From the results of the previous literature, the therapeutic effect of KT on muscle contractile performance was controversial as some studies demonstrated beneficial effects, while others did not [8,22]. Kim and Lee observed significant differences in both flexor and extensor peak torque at the angular velocities of 60°/sec and 180°/sec in horse racing jockeys without knee problems after KT application [8]. On the contrary, Wageck et al. did not find a significant difference in the flexor or extensor peak torque at 60°/sec between the KT-treated and sham-taping group after four days of taping in knee OA patients [22]. Conflicting results may be due to inconsistencies in study populations and taping techniques [9]. Therefore, in the present meta-analysis, we aimed to investigate only the effects of KT in OA patients, and all taping procedures used in the studies included a Y- or I-strip applied to the quadriceps [3,4,6,15,19,20,22,23,24,25,26,27]. The effect of KT on increasing muscle strength should be more significant in patients with chronic musculoskeletal diseases such as OA than in people without disability [15]. The previous literature agrees that KT can reduce pain [14,27,29]; thus, the patient might be able to push harder on the strength measuring device with less pain. Moreover, a reduction in knee pain reduces the pain-induced muscle inhibition of the quadriceps, which has been found to cause selective quadricep weakness seen in subjects with knee OA [19]. Decreased pain might also lead to increased physical activity, and further improved muscle strength in knee OA patients [31].

Unlike previous studies, we separated the muscle strength data into two subgroups: measurements with an isokinetic dynamometer and measurements with an isometric dynamometer by using an HHD. Interestingly, we found a significant moderate total effect favoring the KT group in the isokinetic subgroup but not in the isometric subgroup. Three possible reasons could have caused this result. First, the diverse properties of the two strength measuring devices could cause this difference, as isometric strength measured by an HHD was less accurate and had greater variation. Although HHD measured strength has been proven to have a moderate to high correlation with isokinetic dynamometry, it is still thought to be greatly affected by rater-related factors, such as the strength and proficiency of the rater, as well as the position and fixation of the subject [32]. In contrast, the measurement of peak torque with an isokinetic dynamometer involves a standard procedure and is often used as the gold standard for force measurement [33]. Second, biomechanically, according to the length–tension relationship, muscles can produce maximum tension only when they are at the optimal (resting) length. The active tension decreases when the muscles are excessively elongated or shortened. Previous studies confirmed that KT can not only change the passive tension of connective tissues but can also alter the length of muscles [34]. The isokinetic dynamometer measures the peak torque produced by the muscle under a fixed angular velocity, so the muscle must resist the dynamometer at different angles. We infer that KT can assist muscle contraction at different angles; as a result, there was a significant improvement in peak torque in the KT or KT plus conservative treatment group. Third, only two studies were included for isometric muscle strength analysis, and each measured different muscles of the lower limbs. This can lead to increased heterogeneity and may be the reason that a non-significant total effect was found in the isometric muscle strength meta-analysis.

### 4.3. Non-Significant Effect in Function and ROM

A non-significant total effect was found in the time to complete functional tasks from the data of four studies [6,23]. Anandkumar et al. measured the time to complete a SSCT, which required the patient to ascend and descend a flight of stairs as fast as possible. A large effect size was found in the study of Anandkumar et al. in favor of the KT group [6]. On the other hand, in Mutlu’s study, three timed functional tasks were assessed, including walking 8 m, ascending and descending seven steps, and travelling a distance of 2 m to reach a chair and sit down. The patients in the study of Mutlu et al. were asked to perform the tasks at their own comfortable pace. Only the walking task revealed a significant difference compared with the placebo-taping group [23]. The non-significant finding in the present meta-analysis might have been caused by inconsistencies in task instructions, and it may be speculated whether the task measurement at an individual’s natural pace is suitable for detecting changes in the function of OA patients. A meta-analysis by Lu et al. stated that KT can improve function in knee OA patients, according to improvement in the WOMAC total score [16]. However, the WOMAC is composed of three subscales, including pain, stiffness, and physical function, which is used to evaluate the overall condition of OA patients, not only the function. It is not appropriate to take this index to represent the function of OA patients alone. Future studies may consider measuring the maximum speed for completing functional tasks as an outcome variable for evaluating knee OA patients. The improvement in isokinetic strength and pain reduction might show a greater contribution to increasing the maximum speed rather than the comfortable speed.

The reduction in ROM in knee OA may result from joint stiffness, pain, and kinesiophobia, i.e., pain-related fear of movement [2]. KT treatment emphasizes the elastic property of the tape to assist muscle contraction and thus increase joint mobility. In addition, the support and sensory feedback provided by the tape can reduce kinesiophobia [5]. However, according to the literature, only a few studies found significant small beneficial effects on ROM improvement after applying KT [3,24], while other studies did not [4,23]. The disparity of data collected in different studies might have caused the diverse findings and the non-significant total effect in the present meta-analysis. Although all data included active ROMs, different motions of the lower extremity were included. Three studies measured knee flexion and extension [4,24,27]. The study of Mutlu et al. measured the ROMs of the lower extremity related to functional tasks of daily life, including hip internal and external rotation, knee flexion, and extension [23]. Cho et al. and ÖĞÜT et al. measured changes in the pain-free ROM of the knee rather than in individual motions [3,25]. Moreover, in the study of Cho et al., a significant large effect size was shown for KT treatment over placebo treatment, and a significant correlation was found between pain-free ROM and pain [3]. This finding might imply that pain-free ROM is an indicator that facilitates the observation of changes in knee OA patients.

### 4.4. Limitations

There are several limitations in this study. First, due to a lack of current literature with clinically pertinent outcomes on the effect of KT treatment for OA patients, only 11 RCTs with a Jadad score higher than 3 were included in the meta-analysis, and they did not evaluate identical outcome variables. There is potential for bias in the validity of our results. Second, if multiple data representing the same outcome of one study were included in the meta-analysis, the weight of that study would increase in proportion. As a result, the total effect would deviate towards the study with more outcome data. Third, there is no specific technique for KT application, and it usually varies according to the symptoms of the patient, therapist’s experience, and intended purpose. This heterogeneity, such as different tensions of the tape or applying KT in different directions and shapes, may have caused the inconsistent results and led to non-significant total effects. Finally, KT is not typically used as a single treatment tool but is instead combined with other treatments, such as physical therapy and exercise therapy. This approach is consistent with Dr. Kase’s theory that KT is mainly meant to assist the patient’s movements via its elastic properties [5,7,8]; therefore, the technique with which the tape is applied and the treatment with which it is combined will also affect the results.

## 5. Conclusions

The present meta-analysis showed that KT or KT plus conventional therapy has significant effects on pain relief and isokinetic but not on isometric muscle strength improvement for patients with knee OA. No significant effects were found in HHD-measured isometric muscle strength improvement, time to complete functional tasks, or ROM increase. The finding of increased isokinetic muscle strength is particularly valuable as muscle strength is very important for knee stability [1]; thus, KT can play an important role in rehabilitation. This study supports KT as an effective tool for clinicians or physical therapists in treating knee OA. However, due to the significant heterogeneity shown in all outcome variables and the different application methods of KT, the findings should be interpreted with caution. Future RCTs can consider using isokinetic muscle strength, the fastest speed for functional tasks, and pain-free ROM for their outcome measurements after treating knee OA patients with KT. Moreover, the method of KT treatment should be described in more detail, e.g., the direction of application and the amount of tension used. The effect of KT on other degenerative musculoskeletal diseases is an issue that warrants further study.

## Figures and Tables

**Table 1:** Study characteristics of KT interventions in knee OA patients.

Author (Year)	Study Design	Participants	Intervention	Outcome Measures	Evaluation Time	Results	Jadad Score
Anandkumar et al. [6] (2014)	RCT	40 subjects (17 males; 23 females) with knee OA randomly allocated to experimental group (n = 20) or control group (n = 20)	Experimental group: Three I-shaped KT strips applied from origins of the RF, VL and VM muscles to insertions with 50~70% tension. Control group: Same as the experimental group but with no tension.	1. Pain during SSCT (VAS) 2. Peak isokinetic quadriceps torque 3. Functional task: SSCT 4. ROM: N/A 5. Other: N/A	Before tx, 30 min after KT application	A large effect sizewith significant improvements in pain, peak quadriceps torque, and SSCT was obtained in the experimental group compared to the control group immediately after KT application.	5
Donec and Krisciunas [4] (2014)	RCT	89 post-TKR patients (13 males; 76 females) randomized to experimental group (n = 40) or control group (n = 49)	Experimental group: Postop rehabilitation program and KT, including lymphatic correction, Y strip for RF facilitation (paper-off tension) and I strip for medial knee ligaments. Control group: Only postop rehabilitation program.	1. Postop pain (NPRS) 2. Muscle strength: N/A 3. Functional task: N/A 4. ROM 5. Other: Edema (leg circumference at 4 standardized points)	Before tx and 2^nd^, 8^th^, 16^th^, 24^th^, and 28^th^ days postop	In both groups, postop pain decreased significantly; however, less pain was found in the KT group from the 2^nd^ week. Knee extension was better in the experimental group on the 28^th^ day postop. Edema was less intense and subsided more quickly in the KT group.	3
Donec & Kubilius [19] (2019)	RCT	157 patients (33 males; 124 females) with knee OA randomized to KT group (n = 81) or control group (n = 76)	Experimental group: Two Y strips with paper-off tension, one applied from RF toward the patella, the other from the tibial tuberosity to VM and VL. Two I strips with 75–100% tension, applied on the patella tendon and the collateral ligamnets. Control group: Sham tape applied transversely over the thigh, calf, medial, and lateral sides of the knee joint.	1. Pain (NPRS) 2. Muscle strength: N/A 3. Function: N/A 4. ROM: N/A 5. Other: PPT, KOOS	Before tx, 1 month (immediately after tx), 1 further month after tx (follow-up)	All self-reported improvement remained at the 1-month follow up. Significantly higher and clinically meaningful reduction of pain intensity was found in the KT group after the treatment month, in comparison with the control group. More pain reduction was reported in the daytime for participants in the KT group at the follow up.	5
Kocyigit et al. [20] (2015)	RCT	41 patients (13 males; 28 females) with knee OA randomized to experimental group (n = 21) or control group (n = 20)	Experimental group: Y-shaped KT applied from the RF, with ends around the patellae (25% tension). Y-shaped KT from the tibial tuberosity, with ends around the patellae (25% tension). I-strip KT applied mediolaterally to the patella. Control group: Sham taping with 5 cm surgical hypoallergenic flexible tape with no tension.	1. Activity & nocturnal pain (VAS) 2. Muscle strength: N/A 3. Functional task: N/A 4. ROM: N/A 5. Other: Lequesne index, QOL (NHP score).	Before tx, after 12 days of tx	In both groups, VAS for activity pain, VAS for nocturnal pain, Lequesne index score, and NHP score decreased significantly. NHP energy scores were significantly different between the groups in favor of sham taping.	5
Cho et al. [21] (2015)	RCT	46 patients (13 males; 33 females) with knee OA randomized to experimental group (n = 23) or control group (n = 23)	Experimental group: Y-shaped KT starting from the originof RF to the insertion with 15–25% tension. Control group: Placebo KT applied in the same way with no tension.	1. Pain (VAS) 2. Muscle strength: N/A 3. Functional task: N/A 4. Pain-free AROM 5. Other: PPT (midportion of the quadriceps and TA), Proprioception.	Before tx, 1 hour after KT application	The experimental group showed attenuation of pain during walking, decreased PPT, and significantly improved AROM and proprioception. There were significant differences in pain during walking and PPT between the 2 groups.	5
Wageck et al. [22] (2016)	RCT	39 patients (5 males; 34 females) with knee OA randomized to experimental group (n = 19) or control group (n = 20)	Experimental group: 3 KT application techniques, including drainage, quadriceps relaxation with paper-off tension (Y strip), and pain relief (star application). Control group: Sham taping with two I-shaped KT strips applied without tension across the quadriceps.	1. Pain: N/A 2. Knee extensor and flexor isokinetic torque 3. Functional task: N/A 4. ROM: N/A 5. Other: PPT, Volumetry, Perimetry, Lysholm score, WOMAC.	Before tx, after 4 days of tx, 19 days after tx (follow-up)	On day 4 and 19, there were no significant between-group differences in knee extensor/flexor muscle strength, PPT at any measured point, volumetry, perimetry, Lysholm score, or WOMAC score.	5
Mutlu et al. [23] (2017)	RCT	39 patients (6 males; 33 females) with knee OA randomized to experimental group (n = 20) or control group (n = 19)	Experimental group: Two Y-shaped KT strips applied to the RF and hamstring muscles from origin to insertion with 25% tension. Control group: Placebo KT applied transversely to the muscle groups of the quadriceps and hamstrings at 2 levels.	1. Pain (VAS)2. Isometric muscle strength (HHD) 3. Functional task: ALF (walking 8m; up &down 7 steps; transferring) 4. Active ROM 5. Other: WOMAC.	Before tx, immediately after tx, 6–9 days after tx, 1-month follow-up	The KT group had a large decrease in VAS activity and walking task scores from the initial taping application to 6–9 days after tx to the 1-month follow-up compared with the control group. No significant difference in outcome measures for ROM and muscle strength between groups.	5
Aydogdu et al. [24] (2017)	RCT	54 patients (8 males; 46 females) with knee OA randomized to experimental group (n = 28) or control group (n = 26)	Experimental group: Conventional tx and Y-shaped KT strips applied to the RF and hamstring muscles from origin to insertion with 50–70% tension. Control group: Conventional tx including physical agents (hot pack, USD, TENS) and supervised exercises (stretching and strengthening exercises).	1. Pain (VAS) 2. Isometric muscle strength (HHD) 3. Functional task: N/A 4. ROM 5. Other: KOOS.	Before tx, 1 hour after taping in first tx session, 3 weeks after tx started	Significant differences were observed in pain, ROM, and functional status (KOOS) between pre-tx and post-taping in the experimental group.	3
ÖĞÜT et al. [25] (2018)	RCT	61 patients (females) with knee OA randomized to experimental group (n = 31) or control group (n = 30)	Experimental group: Conventional tx and 3 Y-shaped KT strips applied to the RF (paper-off tension), VL (full stretching at the ends), and VM (full stretching at the ends) from origin to insertion for facilitation.Control group: Conventional tx (hot pack, USD, TENS) and sham taping (I strip applied transversely over quadriceps muscle).	1. Pain (VAS) 2. Knee extensor isokinetic torque 3. Functional task: 50-m walking time 4. ROM 5. Other: WOMAC	Before tx, immediately after tx, 1-month follow-up, 3-month follow-up	In the KT group, the WOMAC pain and WOMAC total scores decreased significantly after treatment compared to the sham-KT group. In both groups; VAS, WOMAC pain, stiffness, physical function, and total values decreased significantly after treatment. In both groups, isokinetic quadriceps peak torque measurements were increased after treatment.	5
León-Ballesteros et al. [26] (2018)	RCT	32 patients (females) with knee OA randomized to experimental group (n = 16) or control group (n = 16)	Experimental group: Quadriceps strengthening exercise program and 2 KT strips (I and Y-shape) applied from 15 cm above the interarticular line to below with no tension.Control group: Quadriceps strengthening exercise program and sham tape (I strip with >50% tension).	1. Pain (VAS) 2. Muscle strength: N/A 3. Functional task: N/A 4. ROM: N/A 5. Other: WOMAC	Before tx, end of the weeks 2, 4, and 6 (end of tx).	At the end of the tx, there were no significant differences in pain between the groups. Both groups had a difference of 2.7 points with respect to the baseline measurement, change percentage of 32.2% and31.1% for placebo and experimental respectively.	5
Rahlf et al. [27] (2018)	RCT	141 patients (67 males; 74 females) with knee OA randomized to KT group (tx group) (n = 47) or sham tape group (control group) (n = 47), and no tape group (n = 47).	Experimental group: One I strip over the patella from the tibial tuberosity to the lower third of the quadriceps with max. tension. 2 I strips over medial and lateral collateral ligaments, respectively. Control group: Sham tape applied transversely distal to the knee with no tension.	1. Pain: N/A 2. MVIC measured by isokinetic dynamometer 3. Functional task: 10 meter walk test 4. ROM 5. Other: WOMAC, BESS	Before tx, immediately after tx (tape applied for 3 consecutive days)	Significant effects were found for WOMAC pain (tape vs. sham; tape vs. control, stiffness (tape vs. sham; tape vs. control), and physical function (tape vs. sham; tape vs. control). No interactions were found for balance, muscle strength, walking speed, or active range of motion.	5

※RCT = randomized control trial, tx = treatment, VAS = visual analog scale, NPRS = numeric pain rating scale, RF = rectus femoris, VL = vastus lateralis, VM = vastus medialis, TA = tibialis anterior, HHD = handheld dynamometer, MVIC = maximal voluntary isometric contraction, SSCT = standardized stair climbing task, ROM = range of motion, PPT = pressure pain threshold, QOL = quality of life, NHP = Nottingham Health Profile, ALF = aggregated locomotor function, WOMAC = Western Ontario and McMaster Universities Arthritis Index, K-WOMAC = Korean version of WOMAC, KOOS = knee injury and osteoarthritis outcome score, BESS = balance error scoring system, USD = ultrasound, TENS = transcutaneous electrical nerve stimulation, N/A = not available.

## Data Availability

Data supporting the reported results can be found in the PubMed, Ovid Medline, CINAHL, Airiti Library, EMBASE, and WOS databases, all of which can be publicly accessed.
